# Time for the 70 °C water precautionary option in the home dilution of powdered infant formula

**DOI:** 10.1186/s13052-016-0228-9

**Published:** 2016-02-19

**Authors:** Marco Silano, Paola Paganin, Riccardo Davanzo

**Affiliations:** Unit of Human Nutrition and Health, Department of Veterinary Public Health and Food Safety, Istituto Superiore di Sanità, Viale Regina Elena 299, 00161 Rome, Italy; School of Pediatrics, University of Trieste, Trieste, 34100 Italy; Division of Neonatology, Institute for Maternal and Child Health - IRCCS “Burlo Garofolo”, Via dell’Istria 65/1, Trieste, 34100 Italy

**Keywords:** Formula preparation, *Cronobacter sp.* infection, Home dilution, Infant sepsis

## Abstract

Powdered infant formulas (PIF) are usually not sterile and may frequently be contaminated by several bacteria strains. Among them, *Cronobacter species*, previously known as *Enterobacter sakazakii*, is one of the most harmful, since it might be the causative agent of sepsis and meningitis in newborns and preterm infants during the first weeks of life. The mortality rate of these infections is up to 80 %. Therefore, some precautions are required in the home handling and dilution of PIF. Whereas there is wide consensus about the need that a PIF should be used immediately after being diluted or, if not, stored at < “5 °C”, still recently the optimal temperature of the water used to dilute PIF is controversial among scientific societies and health agencies. The current knowledge is reviewed in this paper and provides sufficient evidence to cautiously advise the use of hot water at a temperature of “70 °C” in the dilution of PIF in order to prevent the *Cronobacter sp.* contamination and growth.

## Background

The Gram-negative *Cronobacter species* (also known as *Enterobacter sakazakii*) has been demonstrated to be one of the most frequent causative agent of sepsis and meningitis of the newborn [1]. The main route for the neonatal *Cronobacter sp.* infection is by means of the contamination of powdered infant formula (PIF) [2]. Contamination prevalence of powdered milk cans has been reported to range from 6.6 % in Brazil to 29 % in China [3, 4]. However, the great majority of the cases reported worldwide are from five countries only: USA, UK, France, Belgium and Philippines [5].

Although this high rate of PIF contamination by *Cronobacter sp*., in most cases the bacterial load found was low [6] and the prevalence of neonatal infection resulted small. In USA, it has been reported 1 case out of 100.000 infants under a year of age, figure that rises to 8.7 among low birth weight infants and further to 9.3 among very low birth weight infants [6]. The British Health Protection Agency has reported in the period 1992 – 2002, only 16 cases of *Cronobacter sp.* positive blood/CSF culture among newborn infants (<1 month of age) and 20 for those infants aged between 1 and 11 months [7]. In the Netherlands, *Cronobacter sp.* infections are estimated to represent 0.5–2.4 % of foodborne infectious diseases and roughly 0.7 % of the overall meningitis [8]. On the contrary, the overall mortality of *Cronobacter sp.* infections is very high, up to 80 % [5].

So far, no case of *Cronobacter sp.* infection via powder milk has been reported in Italy among the newborns, although *Citrobacter freundii*, a pathogen known to cause diarrhea among infants [9] and severe neonatal infection [10] has been isolated in 32 samples of PIF out of 75 examined [11].

Beside *Cronobacter sp*., other bacteria could contaminate PIF. Between 1985 and 2005 at least 6 outbreaks of salmonellosis, involving as many as 250 infants, have been associated with contaminated PIF [12]. Moreover, *Clostridium botulinum* type B was isolated from an opened container of a PIF can from the home of a 5-months-old infant with confirmed botulism, and from an unopened container of the same batch obtained prior to distribution and retail sale. The PIF consumed by the patient was from a batch in the meantime recalled by the manufacturer [13].

The contamination of PIF by *Cronobacter sp.* is a global issue and an area of dispute, because it isn’t clear in which phase of PIF production and storage the contamination can occur. Moreover, *Cronobacter sp.* has been isolated with varying frequency in production environments from food (milk powder, chocolate, cereal, potato, and pasta) factories and households, strongly indicating that it is widespread [14].

It is usually agreed that *Cronobacter sp.* is relatively thermo resistant, so it can survive after pasteurization [15] and that contamination by *Cronobacter sp.* could occur during the reconstitution procedures of PIF.

## Recommendations on home preparation of PIF in order to prevent Cronobacter sp. infections

A clear consensus on the recommendation for home preparation of PIF is lacking and several contradictory guidelines aimed at PIF contamination are available.

The U.S. Food and Drug Administration gives the following advice [16]: “Standards for manufacturers of infant formula [*Omissis*] include: Current good manufacturing practices specifically designed for infant formula, including required testing for the harmful pathogens [disease-causing bacteria] *Salmonella* and *Cronobacter*. [*Omissis*] In most cases, it’s safe to mix formula using ordinary cold tap water that’s brought to a boil and then boiled for one minute and cooled.” The former advice “According to the World Health Organization, recent studies suggest that mixing PIF with water at a temperature of at least “70 °C” —“158 °F”— creates a high probability that the formula will not contain the bacterium *Enterobacter s.* — a rare cause of bloodstream and central nervous system infections,” is no longer indicated.

More in depth, the U.S. Center for Disease Control [17] concluded that: “*Cronobacter* bacteria can cause severe blood infections or meningitis. Infants of 2 months of age and younger are most likely to develop meningitis if they are infected with *Cronobacter* bacteria. Infants born prematurely and those with weakened immune systems are also at increased risk for serious *Cronobacter* infections. [*Omissis*] In some outbreak investigations, *Cronobacter* bacteria were found in PIF that had been contaminated in the factory. In other cases, *Cronobacter* bacteria might have contaminated the PIF after it was opened at home or elsewhere during preparation. [*Omissis*] Manufacturers report that, using current methods, it is not possible to eliminate all germs from PIF in the factory. PIF can also be contaminated after the containers are opened. Very young infants, infants born prematurely, and infants with weakened immune systems are at the highest risk. [*Omissis*] If your baby gets formula, choose infant formula sold in liquid form, especially when your baby is a newborn or very young. Liquid formulations of infant formula are made to be sterile and should not transmit *Cronobacter* infection. [*Omissis*] If your baby gets PIF, there are things you can do to protect your baby from infections – not just *Cronobacter* infections. Good hygiene, mixing the formula with water hot enough to kill germs, and safely storing formula can prevent growth of *Cronobacter* bacteria and other germs. These are keys to keeping your baby safe and healthy. [*Omissis*] Use hot water (158 °F/70 °C and above) to make formula. [*Omissis*] Use formula within 2 h of preparation. If the baby does not finish the entire bottle of formula, discard the unused formula. If you do not plan to use the prepared formula right away, refrigerate it immediately and use it within 24 h.

The position of the British National Health System is quite similar to the previous ones [18]: “Use freshly boiled drinking water from the tap to make up a feed. Don’t use artificially softened water or water that has been boiled before. Leave the water to cool in the kettle for no more than 30 min. This will ensure it stays at a temperature of at least 70 C. Water at this temperature will kill any harmful bacteria.”

The European Food Safety Agency recognizes the compelling evidence that consumer categories vulnerable to *Enterobacter s.* are premature and low birth weight infants and those aged <28 days [19]. Nevertheless, no clear recommendation is provided about the appropriate temperature for PIF home dilution in order to prevent *Cronobacter sp.* infections [20]. The European Commission has not dealt with this topic in the Regulation 1169/2011 on labeling of the foodstuff.

The European Society of Pediatric Gastroenterology, Hepatology and Nutrition does not consider necessary the use of water with a temperature > 70 °C for the dilution of PIF. In fact, the main concern of the ESPGHAN experts is that the hot water may inactivate the vitamins and kill the probiotic strains contained in the formula [21].

Since powdered milk-borne *Cronobacter sp*. infections have not been reported in Italy so far, the Italian Ministry of Health has not been urged to express an official position on this topic. As well, the Italian Society of Pediatrics and the Italian Society of Neonatology do not have official positions on this issue.

## Conclusions

There is a wide consensus on WHO/FAO guidelines concerning the need that PIF should be used immediately after being diluted or, if not, stored at < “5 °C” [22]. Notwithstanding the current controversies on the home dilution of PIF, current scientific evidence suggests a precautionary approach. We therefore believe that it is advisable the use of “70 °C” warm water for the dilution of PIF, especially for feeding preterm and low birth weight infants. In our opinion, it is not worthy performing a routinary analysis of the eventual presence of *Cronobacter sp.* in PIF neither in Neonatal Intensive Care Units nor at home, since the use of water at “70 °C” for the dilution is efficient in preventing with high probability, the presence and the growth of live strains of *Cronobacter sp.*

In the meantime, baby food industry is challenged to reduce the level of *Cronobacter sp.* contamination of PIF, also experimenting new technologies able to inactivate the *Cronobacter sp*. while limiting the disruption of vitamins and other important nutrients. Researchers are investigating methods to improve the microbiological safety of PIF, via the inclusion of protectants or alternative methods to lower or eliminate microbiological contamination [23]. Among the most promising, bioactive peptides, organic acids, probiotics and prebiotics should be cited [23, 24]. Supercritical cabon dioxide, gamma irradiation, exposure to microwaves or to a combination of UV and near infrared radiation heating are some among the possible alternative physical methods [23, 25].

Eventually, all these new technologies, though promising, still need further investigation on safety, reproducibility and cost/benefit ratio issues, before becoming standard procedures.

## Ethics approval

This article does not contain any studies with human participants or animals.
